# Nutritional status of HIV-infected patients during the first year HAART in two West African cohorts

**DOI:** 10.1186/s41043-015-0001-5

**Published:** 2015-05-01

**Authors:** Maryline Sicotte, Chantal Bemeur, Assane Diouf, Maria Victoria Zunzunegui, Vinh-Kim Nguyen, for the ATARAO initiative

**Affiliations:** 1 Research Center of the Montreal University Hospital Center (CRCHUM), Montreal, Canada; 2 School of Public Health, Montreal University, Pavilion 7101 Parc Avenue. C.P. 6128, Succ. Centre-Ville, Montreal, QC H3C 3 J7 Canada; 3 Department of Nutrition, Montreal University, Montreal, Canada; 4 Deparment of Infectious Disease, Fann’s University Hospital Center, Dakar, Senegal; 5 Montreal University Public Health Research Institute (IRSPUM), Montreal, Canada; 6 Department of Social and Preventative Medicine, Montreal University, Montreal, Canada; 7 Fann University Hospital Center, and School of Public Health, Montreal University, Montreal, Canada

**Keywords:** Malnutrition, Body mass index (BMI), Hemoglobin, Albumin, CD4, Highly active antiretroviral treatment (HAART), Low- and middle-income countries (LMIC)

## Abstract

**Objective:**

To examine the association between nutritional markers at initiation and during follow up in two different cohorts of HIV-infected adults initiating highly active antiretroviral therapy (HAART) in West Africa.

**Methods:**

The ATARAO study was a one year prospective study carried in Mali. It consisted of a sample of consecutive patients initiating HAART in one of four participating centers during that period. Data were collected at time of treatment initiation (baseline) and every 3 months thereafter. The ANRS 1290 study followed Senegalese patients recruited in similar conditions. Bivariate analyses were used to identify nutritional and immunological covariates of malnutrition at baseline. Longitudinal trajectories of body mass index, hemoglobin and albumin, and their associated factors, were evaluated using mixed linear models.

**Results:**

In ATARAO, 250 participants were retained for analyses; of which, 36% had a BMI < 18.5 kg/m^2^, nearly 60% were anemic and 47.4% hypoalbuminemic at time of treatment initiation. At baseline, low hemoglobin, hypoalbuminemia and low CD4 levels were associated with a BMI < 18.5 kg/m^2^. Similarly, low BMI, low albumin and low CD4 counts were linked to anemia; while, hypoalbuminemia was associated with low hemoglobin levels and CD4 counts. In ANRS, out of the 372 participants retained for analyses, 31% had a low BMI and almost 70% were anemic. At baseline, low BMI was associated with low hemoglobin levels and CD4 counts, while anemia was associated with low CD4 counts and female sex. While treatment contributed to early gains in BMI, hemoglobin and albumin in the first 6 months of treatment, initial improvements plateaued or subsided thereafter. Despite HAART, malnutrition persisted in both cohorts after one year, especially in those who were anemic, hypoalbuminemic or had a low BMI at baseline.

**Conclusion:**

In ATARAO and ANRS, malnutrition was common across all indicators (BMI, hemoglobin, albumin) and persisted despite treatment. Low BMI, anemia and hypoalbuminemia were associated with attrition, and with a deficient nutritional and immunological status at baseline, as well as during treatment. In spite of therapy, malnutrition is associated with negative clinical and treatment outcomes which suggests that HAART may not be sufficient to address co-existing nutritional deficiencies.

## Background

In sub-Saharan Africa (SSA), malnutrition is endemic and affects 12-26% of the populations of Mali and Senegal [1]. The term ‘malnutrition’ refers to undernutrition and/or overnutrition. However, in the present study, we focused on malnutrition as a state of undernutrition. Malnutrition results from two major processes, insufficient dietary intake - namely undernutrition - and/or inflammatory activity [2]; that both compromise immune functions [3,4]. In SSA, the HIV epidemic has been surperimposed onto prevalent nutritional deficits. The infection is also a cause of malnutrition through dietary reduction, nutrient malabsorption, inflammation and metabolic disturbances. When coupled, undernutrition and HIV induce a vicious cycle that hastens disease progression and increases mortality [5]. Despite increased access to HAART, poor nutritional status persists [6] and continues to be associated with negative health outcomes [7-9].

Low BMI reflects low energy stores and chronic energy deficiency [10,11]. Between 19-63% of patients living with HIV/AIDS (PLA) in SSA display a low BMI at HAART initiation [9,12-14]. Low BMI strongly predicts disease progression, therapeutic failure and mortality [12,14,15]. More importantly, in low and middle income countries (LMIC) weight loss in the first months of treatment is associated with increased side effects [16,17], poor treatment outcomes and mortality [7,9,13,15].

In PLA, anemia is highly frequent, affecting up to 95% of those in advance stages of the disease [15,18]. Its etiology is multifactorial, HIV-associated cytokine production and viral activity lead to reduced hematopoiesis [19]. In addition, the infection aggravates micronutrient deficiencies that cause anemia [20]. In treated patients, anemia has been associated with excess mortality, poor treatment outcomes [18,21] and excess side effects [22].

In LMIC, up to 55% of PLA suffer from hypoalbuminemia [23-25]. Albumin is a negative acute phase protein (APP) and biological marker of nutritional status [11]. Its synthesis and decline are closely linked to protein and energy intake adequacy, as well as ongoing inflammation consequent to malnutrition and/or HIV infection [26]. Due to its correlation with CD4, both pre and post ART initiation, and its association with mortality despite HAART [23,25,27], hypoalbuminemia has been proposed as a marker of disease progression and treatment response in LMIC [28-30]. Furthermore, albumin may influence the pharmacokinetics [31,32] and exacerbate the side effects of ART [16].

Despite a high prevalence of malnutrition in treated PLA in LMIC, few studies have described post HAART nutritional trajectories in these patients. In two different West African contexts, we examined malnutrition amongst individuals in their first year of treatment by investigating the association between nutritional markers at treatment initiation by and examining nutritional trajectories post HAART.

## Methods

### Study design and population

*ATARAO* (*Appuyer le Traitement AntiRétroviral en Afrique de l’Ouest*) was a one-year cohort that aimed at identifying the determinants of therapeutic success in treatment naïve PLA at two hospitals and two community centers in Bamako and Sikasso, Mali. Attending physicians at participating sites consecutively invited all patients who qualified for ART initiation during the recruitment period to join the cohort. Details concerning inclusion criteria are contained in Table 1. Participants were interviewed at baseline (time of HAART initiation) and invited to return every 3 months thereafter. Informed consent was obtained at baseline. A compensation of ≈ 4 US$ for transportation was given at every interview. This study was approved by Malian *(Health Ministry)* and Canadian *(Montreal University Hospital Centre – CHUM)* ethics committees.

Detailed methodology of the ANRS 1290/1215 *(Agence Nationale de Recherche sur le Sida)* has been described elsewhere [33]. Briefly, the ANRS study is a cohort of patients who initiated HAART as part of the Senegalese antiretroviral drug access initiative (ISAARV) in Dakar (Table 1). Its objectives were to assess the clinical and social impact of HAART. Following pre-enrollment and enrollment visits (baseline), patients were invited to return one month later and every 2 months thereafter. The study was approved by the Senegalese national ethics committee. All patients provided informed consent. The authors of the present article were granted access to the ANRS database, but were not implicated in the study. Collaboration between ANRS and ATARAO began once patient follow-up and databases were completed.

### Data collection

Demographic data were collected at baseline. Every 3 months, BMI was measured by the same staff member (ATARAO) or by the treating physician (ANRS). Blood samples were collected at each trimester for albumin assessment (ATARAO only). Hemoglobin and CD4 values were measured every 6 months in both studies.

### Lost to Follow-Up (LTFU)

Participants were considered LFTU if they missed ≥ 2 consecutive visits (ATARAO) or if they could not be contacted for ≥ 6 months (ANRS). Efforts taken to locate patients, determine their vital status and causes of death of ANRS LTFU patients are described elsewhere [33]. Similar, but less exhaustive methods, were used in ATARAO.

### Outcomes and determinants

*BMI :* Body weight was measured in grams and height was measured to the nearest 0.5 cm using a stadiometer. BMI cutoffs of 18.5 kg/m^2^ and 16 kg/m^2^ were used to identify mild and severely malnourished patients, respectively [34].

*Albumin:* Albumin was only available in ATARAO*.* Albumin was measured in Canada in frozen plasma samples collected in Mali. Albumin levels were estimated using QuantiChrom™ BCG Albumin Assay Kit (*BioAssay Systems*). Albumin values under 3.5 g/dl and <2.5 g/dl indicate moderate and severe hypoalbuminemia, respectively [35].

*Hemoglobin :* Hemoglobin was measured locally by automated hematology analyzer. Because > 95% of patients were at least mildly anemic, we chose to use a more severe definition of anemia. Consequently, a cutoff value of ≤ 11 g/dl was used. According to World Health Organization (WHO), this cutoff identifies individuals with moderate and severe anemia [36].

*CD4 :* CD4 cell counts were measured by FACS count (*Flow Cytometry Assay*) (Becton Dickinson) locally. Only CD4 values measured within a month of the interview’s date were kept for analyses.

### Statistical analyses

Tests for linear trends in proportions were carried out to identify covariables of malnutrition at time of treatment initiation. Mixed linear models were used to examine longitudinal changes in BMI, hemoglobin and albumin. In these equations, the intercept represented the baseline value of the dependent variable and the time coefficient indicated its rate of change in time. A significant quadratic time term indicated the presence of a nonlinear accelerating pattern. The intercepts, linear and quadratic time terms were examined, and treated as random if significant. All models were adjusted for age, sex, time since entry in the cohort, as well as variables found to be associated with the nutritional status at baseline (p < 0.20). Continuous variables were centered to the mean. Interactions between baseline predictors and time were examined. Model selection was done using the *Bayesian Information Criterion* (BIC). For graphic purposes, identical mixed linear models were generated using non-centered variables. Analyses were performed using SPSS 18.

### Missing data

Patients with less than two data entry (including baseline) were excluded from multivariate analyses. Consequently, between 20-34% were excluded from multilevel analyses because of incomplete BMI, hemoglobin or albumin data in ATARAO. In ANRS, 13% of the original cohort were excluded from multilevel analyses because of incomplete BMI or hemoglobin data.

## Results

### Characteristics of patients at baseline

Respectively, 273 and 404 patients from ATARAO and ANRS were initially recruited. Of which, 250 and 372 were retained in analyses (Table 1). During the course of ATARAO, 22 patients died and 82 were LTFU. Deceased ATARAO patients had significantly lower hemoglobin, albumin and CD4 counts at baseline compared to those who survived. Participants LTFU had a lower BMI at baseline compared to those retained. In ANRS, 45 patients died but very few patients were LTFU (n = 7), hence LTFU were not characterized. Deceased ANRS participants had lower BMI, hemoglobin and CD4 in comparison to survivors *(data not shown)*.

**Table 1:** **Studies characteristics**

	**ATARAO**	**ANRS 1215/1290**
*# patients recruited/# patients retained in analyses*	273/250	404/372
*Inclusion criteria (cohort)*	•≥16 years old	•HIV-1 infected
	•Naive to ART	•15 years old
	•Qualified for HAART	•Non-pregnant
	•Reside close to one of the study center	•Qualified for HAART
*Exclusion criteria (analyses)*	•Pregnancy	•Non naive to ART
	•HIV-2 infection or co-infection	
*Start/end date of recruitment*	June 2007/January 2008	August 1998/April 2002
*Length of FU*	1 year	Up to 107 months
*# LTFU(%)/# dead (%)*	82 (30%)/22 (8%)	7 (2%)/45(11%)
*First line regimen*	2 nucleoside reverse transcriptase inhibitors (NRTI) + 1 non-NRTI (NNRTI) (>84% received *Triomune)*	2 NRTIs + 1 NNRTI or one protease inhibitor (41.8%), 4.4% received 2 NRTIs alone before May 2000 [33], 30% received zidovudine
*Nutrition support*	Daily free meals and cooking classes, available to all patients, offered at 1 center.	None

In both cohorts, baseline malnutrition was frequent, irrespective of the indicator analyzed (Table 2). Median BMI was 19.6 kg/m^2^ (IQR: 17.7-22.4) in ATARAO, and 20.0 kg/m^2^ (18.0-22.3) in the ANRS. In both cohorts, a vast proportion of patients suffered from anemia (median Hb = 10.5_(ATARAO)_, 10.6_(ANRS)_; IQR = 9.4-11.8_(ATARAO)_, 9.5-11.9_(ANRS)_). In ATARAO, median albumin level at baseline was 3.67 (IQR = 2.67-5.10). Finally, median CD4 counts were 163 (IQR = 54–275) in ATARAO and 120 (IQR = 46–214) cells/mm^3^ in ANRS. At treatment initiation, mean BMI, hemoglobin and CD4 counts did not differ significantly across studies.

### Covariates of malnutrition

At baseline, the proportion of individuals with a low BMI augmented with worsening of anemia or hypoalbuminemia statuses (Table 3). A similar tendency was observed across strata of CD4 counts. Only in Senegal, women were significantly more anemic than men at baseline. In both cohorts, there was an increasingly greater proportion of patients with hemoglobin levels ≤ 11 g/dl with decreasing BMI and CD4 counts. Albumin levels < 3.5 g/dl were increasingly more frequent across higher grades of anemia and lower CD4 counts. Although not significant, hypoalbuminemia appeared to be more frequent among those with lower BMI.

Pooled data from both cohorts were used to examine associations between nutritional markers (BMI, hemoglobin, and albumin) according to CD4 counts (CD4 < 200, CD4 ≥ 200). In spite of stratification, associations between BMI and hemoglobin maintained their significance *(data not shown)*.

### Baseline determinants of nutritional trajectories

**Figure 1:**
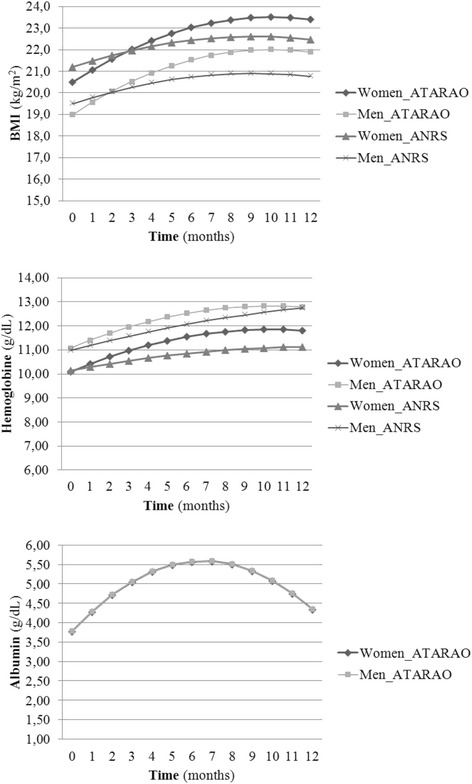
Nutritional trajectories according to average baseline values.

Table 4 presents the associations between baseline nutritional and immunological characteristics, and variations in nutritional trajectories across time for both studies. Figure 1 illustrates BMI, hemoglobin and albumin sex-specific trajectories during follow up.

In model 1, random and fixed linear and quadratic time terms were significant in both cohorts (p < 0.001) indicating a positive change in BMI in the first months followed by a later decline. Women initiated treatment with higher BMI, but gained in a similar fashion to men over time (Figure 1). In ATARAO, BMI trajectories reached a plateau after 9 months of treatment with a slight decrease at the end of the year. In ANRS, men and women gained less over time and plateaued at around the same time as in ATARAO. At 6 months, 12% and 17% of Malian and Senegalese patients, respectively, continued to have a BMI <18.5 kg/m^2^. While this proportion decreased to 6.9% at 12 months in Mali, as many Senegalese (17.8%) were malnourished according to their BMI status at the end of the first year of treatment. Most of these (>70%) had a BMI <18.5 kg/m^2^ at HAART initiation in both populations (*data not shown*).

**Table 2:** **Baseline characteristics of patients from the ATARAO and ANRS 1290 cohorts**

	**ATARAO (n = 250)**		**ANRS (n = 372)**		
	**N**	**%**	**N**	**%**	P value*
**Sex**					**<0.001**
*Men*	74	29.6	168	45.2	
*Women*	176	70.4	204	54.8	
**Age** (years)					**0.001**
*<25*	27	10.8	19	5.1	
*25-29*	45	18.0	57	15.3	
*30-39*	105	42.0	146	39.2	
*≥40*	73	29.2	150	40.3	
**BMI** (kg/m^2^)					0.307
*≤16*	32	12.8	31	9.7	
*16-18.5*	58	23.2	68	21.3	
*18.5-25*	140	56.0	191	59.7	
≥25	20	8.0	30	9.4	
**Hemoglobin** (g/dl)					0.665
<8.0	21	8.5	26	7.0	
8-9.5	43	17.5	66	17.9	
9.5-11.0	79	32.1	114	30.9	
>11.0	103	41.9	163	44.2	
**Albumin** (g/dl)					-
*≤2.5*	52	21.7	-	-	
*2.5-3.5*	63	26.3	-	-	
*3.5-4.2*	38	15.8	-	-	
>4.2	87	36.3	-	-	
**CD4** (cells/mm^3^)					0.116
*<100*	93	37.2	154	42.3	
*100-200*	53	21.2	105	28.8	
*200-350*	86	34.4	86	23.6	
*>350*	18	7.2	19	5.2	

*p value comparing means between cohorts.

Bold data = p < 0.05.

There was an initial improvement in hemoglobin levels subsequent to treatment initiation, followed by a decline in later months (model 2, Figure 1). Respectively, 31 and 33% of patients were anemic at 6 months in ATARAO and ANRS, while 26 and 29% continued to be anemic at 12 months. Women had lower hemoglobin levels in both cohorts. Furthermore, Senegalese women showed a greater decline over time than men. Although age, albumin and CD4 in ATARAO were not significant, model adjustment was improved by keeping these variables (Table 4).

In ATARAO, albumin increased similarly for both men and women during the first 9 months of treatment, but declined in the course of the last trimester (model 3, Figure 1). Respectively, 6% and 11% of patients had albumin level < 3.5 g/dl at 6 and 12 months (*data not shown*). Sex was excluded as it failed to improve the fit of the model.

**Table 3:** **Factors associated with malnutrition at baseline: bivariate associations**

	**BMI < 18.5**			**Anemia**			**HypoALB** ^¥^		
	**N**	**%**	P*	**N**	**%**	**P**	**N**	**%**	**P**
ATARAO									
Sex			0.788			0.055			0.773
*Men*	74	35.1		74	50.0		71	46.5	
*Women*	176	36.9		176	63.1		169	48.5	
Age (years)			0.186			0.531			0.151
*<25*	27	51.9		27	70.4		25	60.0	
*25-29*	45	37.8		45	62.2		45	53.3	
*30-39*	105	32.4		105	52.4		99	44.4	
*≥40*	73	36.4		73	63.0		71	45.1	
BMI (kg/m^2^)						**0.000**			0.052
*≤16*	-			32	75.0		31	53.3	
*16-18.5*	-			58	81.0		55	62.5	
*18.5-25*	-			140	50.0		142	44.8	
≥25				20	35.0		22	41.2	
Albumin (g/dl)			**0.015**			**0.007**			
*≤2.5*	52	50.0		52	71.2		-		
*2.5-3.5*	63	35.5		63	65.1		-		
3.5-4.2	38	31.6		38	60.5		-		
*>4.2*	87	28.7		87	49.4		-		
Hemoglobin (g/dl)			**0.000**						**0.024**
*<8*	21	61.9		-			97	40.2	
*8-9.5*	43	53.5		-			77	49.4	
*9.5-11*	79	41.8		-			41	61.0	
*>11*	103	21.4		-			21	57.1	
CD4 (cells/mm^3^)			**0.001**			**0.002**			**0.009**
*<100*	93	47.3		93	66.7		90	55.6	
*100-200*	53	28.3		53	66.0		50	50.0	
*200-350*	86	25.6		86	54.7		82	45.1	
*>350*	18	16.7		18	22.2		18	16.7	
ANRS									
Sex			0.132			**0.000**			
*Men*	148	35.1		166	47.6		-		
*Women*	172	27.3		203	68.0		-		
Age (years)			0.777			0.305			
*<25*	15	26.7		19	68.4		-		
*25-29*	50	30.0		56	64.3		-		
*30-39*	120	35.0		145	56.6		-		
*≥40*	135	28.1		149	57.7		-		
BMI (kg/m^2^)						**0.002**			
*≤16*	-			31	80.6		-		
*16-18.5*	-			67	68.7		-		
*18.5-25*	-			189	53.4		-		
≥25	-			30	53.3		-		
Hemoglobin (g/dl)			**0.013**						
*<8*	23	43.5		-			-		
*8-9.5*	61	36.1		-			-		
*9.5-11*	99	35.4		-			-		
*>11*	137	23.1		-			-		
CD4 (cells/mm^3^)			**0.017**			**0.000**			
*<100*	136	38.2		154	69.5		-		
*100-200*	88	30.7		104	55.8		-		
*200-350*	71	18.3		85	45.9		-		
*>350*	19	31.6		19	42.1		-		

* Obtained by trend test (linear by linear association chi^2^ statistic).

¥ Albumin (ALB) was only available in ATARAO.

Bold data = p < 0.05.

## Discussion

Our study was the first to investigate BMI, hemoglobin and albumin in a cross-sectional and longitudinal manner among populations initiating HAART. In both ATARO and ANRS, an important proportion of patients were malnourished at time of treatment initiation, irrespective of the nutritional marker investigated. These results coincides with observations from other African studies [7,15,24,25,37]. As in these previous reports, significant covariation between nutritional markers was observed at baseline, which could suggests that these indicators capture a similar process.

As demonstrated here, independent of baseline nutritional status, HAART contributed to early improvement in BMI, hemoglobin and albumin statuses in most patients. Both in ATARAO and ANRS studies, the proportion of patients with a low BMI, anemia or hypoalbuminemia decreased during the first year of treatment – especially in the first 6 months. However, trajectories plateaued during the second part of the year. Consequently, low BMI remained prevalent. Twelve months after initiating HAART, 7% of patients continued to have a BMI < 18.5 kg/m^2^ in ATARAO and 18% in ANRS – 70% of whom had a low BMI at treatment initiation. BMI trajectories resembled those observed in studies from similar context. As observed elsewhere, most patients gained weight before reaching a plateau at 9 [38] or 12 months post HAART [39]. Typically, those who initiated treatment with the lowest BMI gain the most weight. However, despite this increase, it has been shown that these patients failed to catch up to those well-nourished at HAART initiation.

Similarly, in both ATARAO and ANRS, about a third of patients continued to be anemic at 6 and 12 months despite initial improvements; most of whom were already identified as such at baseline *(data not shown)*. These hemoglobin gains were less impressive in terms of absolute increase and sustainability compared to previous reports [37]. Interaction between sex and hemoglobin trajectories in ANRS could be explained by the use of zidovudine, which was not used in ATARAO [40].

Finally, after 6 months of treatment, less than 6% of participants were hypoalbuminemic compared to 48% at baseline. These early improvements in albumin levels could indicate lessening of the HIV associated inflammatory processes in response to HAART’s action on viral replication [41]. However, by the end of the year, initial gains subsided and an increasing proportion of our patients (11%) displayed hypoalbuminemia. This sudden drop could be attributable to various causes including persisting chronic malnutrition [31,32]. Hence, patients with normal albumin would better benefit from ART. No other study has modeled albumin trajectories post HAART.

Our results indicate that malnutrition either subsists or appears despite HAART. Weight or BMI loss, but also the absence of weight gain in the months following treatment initiation, are risk factors of mortality [8], especially among those initiating treatment with a low BMI [7,13]. In PLA, a deficient nutritional status could result from inadequate nutrient intake, but also from ongoing inflammatory process (either secondary to the infection or malnutrition itself). Many questions have been raised concerning the validity and use of nutritional markers in HIV patients [42,43]. Identifying the exact etiology of subnormal nutritional markers in HIV+ individuals from LMIC is a difficult task as malnutrition is surperimposed onto other ubiquitous inflammatory triggers such as malaria, parasites, and tuberculosis leading to excess immune activation in PLA in these settings [27]. However, we suspect that, for most patients, food insecurity and access to nutritious food varied little during follow up and consequently contributed to maintaining malnutrition amongst treated patients.

Associations between nutritional markers persisted even after stratification by baseline CD4 counts, contrary to previous reports from LMIC. These earlier studies observed a positive significant covariation between BMI and hemoglobin/albumin at low, but not at high CD4 counts, suggesting that they were markers of more advanced disease and inflammation, rather than a nutritional marker [42].

**Table 4:** **Associations between nutritional and immune markers at baseline and nutritional trajectory over 12 months follow-up**

	**Model 1. BMI**		**Model 2. Hemoglobin**		**Model 3. Albumin**	
**ATARAO**	***Estimate (95% CI)***	***p value***	***Estimate (95% CI)***	***p value***	***Estimate (95% CI)***	***p value***
Constant	19.1 (18.2; 20.0)	< .001	11.2 (10.8; 11.6)	<.001	3.85 (3.66; 4.05)	< .001
Sex (Female)	1.41 (0.40; 2.42)	.006	- 0.95 (−1.44;-0.47)	<.001	*-*	
Age*	0.06 (0.01 ; 0.12)	.012	0.003 (−0.022; 0.027)	.826	−0.007 (−0.026; 0.012)	.466
BMI*	*-*		0.13 (0.08;0.20)	< .001	0.08 (0.03; 0.13)	.002
Albumin*	0.36 (0.10; 0.61)	.007	0.12 (−0.007;0.242)	.064	*-*	
Hemoglobin*	0.46 (0.23,0.69)	< .001	*-*		0.09 (0.001; 0.196)	.048
CD4*	0.003 (0.000;0.007)	.021	0.001 (−0.000;0.003)	.077	0.001 (−0.000; 0.003)	.096
**ANRS**	***Estimate (95% CI)***	***p value***	***Estimate (95% CI)***	***p value***		
Constant	19.4 (19.0; 19.9)	< .001	11.1 (10.8; 11.4)	< .001	-	
Sex (Female)	1.48 (0.84; 2.11)	< .001	−0.82 (−1.21; −0.43)	< .001	-	
Age*	0.05 (0.02; 0.09)	.002	*0.01 (−0.00; 0,03)*	.121	-	
BMI*	*-*		*0.08 (0.04;0.12)*	< .001	-	
Hemoglobin*	0.46 (0.31; 0.62)	< .000	*-*		-	
CD4*	0.004 (0.002; 006)	.001	*0.002 (0,000; 0,003)*	.007	-	
Sex x time	*-*		-.0.07(−0.11; −0.02)	.003	-	

Only fixed coefficients are present here.

*Centered to the mean.

Finally, immunodeficiency appeared to be strongly linked to malnutrition at time of treatment initiation, but the predictive value of baseline CD4 was somewhat weaker in multilevel modeling. Many conflicting reports have emerged concerning the interrelation between immunity and malnutrition in HAART treated patients. Some have found positive links [29,30,44]; others have failed to uncover significant associations [25,42,45]. A possible explanation could be that the association between nutritional status and CD4 counts is influenced by the range of CD4 values [44] or prevalence of malnutrition. In our study, associations among nutritional markers were similar in those with relatively high (≥200) and with low (<200) CD4 counts. This observation would argue for the independence of the association between nutritional markers at this low end of the CD4 distribution, suggesting a phenomenon that could not be explained by immunosuppression.

### Limitations

The main limitations of our study were 1) attrition, and 2) missing data, especially biochemical and hematological. Both cohorts suffered from high mortality and LTFU rates. Nonetheless, these rates approximated that observed elsewhere in LMIC [46,47]. In these contexts, LTFU is frequent [48] and linked to non-documented mortality, migration, stigma, and side effects [48]. In both studies, attrition was strongly associated with malnutrition and immunosuppression. This phenomenon led to an under-representation of malnourished individuals and probable misrepresentation of nutritional trajectories. Hence, our models are possibly optimistic evaluations of the true nutritional trajectories in treated PLA.

## Conclusion

As observed in ATARAO and ANRS, low BMI, anemia or hypoalbuminemia, at time of initiation or in the first year of therapy, have an impact on the health of patients as they put them at risk of persistent malnutrition, LTFU and mortality. Independently of treatment and level of immunosuppression, malnutrition persisted among patients receiving HAART. Antiretrovirals alone may not be sufficient to overcome malnutrition and death in treated PLA. Pharmaceutical interventions should be complemented, if not preceded, with programs to fight undernutrition and food insecurity.

## References

[1] FAO (2010). The state of food insecurity in the world: Addressing food insecurity in protracted crises.

[2] Soeters PB, Reijven PL, van Bokhorst-de van der Schueren MA, Schols JM, Halfens RJ, Meijers JM (2008). A rational approach to nutritional assessment. Clinical Nutrition.

[3] Calder PC (2013). Feeding the immune system. Proc Nutr Soc.

[4] Cunningham-Rundles S, Ahrne S, Johann-Liang R, Abuav R, Dunn-Navarra AM, Grassey C (2011). Effect of probiotic bacteria on microbial host defense, growth, and immune function in human immunodeficiency virus type-1 infection. Nutrients.

[5] de Pee S, Semba RD (2010). Role of nutrition in HIV infection: review of evidence for more effective programming in resource-limited settings. Food Nutr Bull.

[6] Hadgu TH, Worku W, Tetemke D, Berhe H (2013). Undernutrition among HIV positive women in Humera hospital, Tigray, Ethiopia, 2013: antiretroviral therapy alone is not enough, cross sectional study. BMC Public Health.

[7] Koethe JR, Lukusa A, Giganti MJ, Chi BH, Nyirenda CK, Limbada MI (2010). Association between weight gain and clinical outcomes among malnourished adults initiating antiretroviral therapy in Lusaka. Zambia J Acquir Immune Defic Syndr.

[8] Auld AF, Ekra KA, Shiraishi RW, Tuho MZ, Kouakou JS, Mohamed F (2014). Temporal Trends in Treatment Outcomes for HIV-1 and HIV-2-Infected Adults Enrolled in Cote d'Ivoire's National Antiretroviral Therapy Program. PLoS One.

[9] Maman D, Glynn JR, Crampin AC, Kranzer K, Saul J, Jahn A (2012). Very early anthropometric changes after antiretroviral therapy predict subsequent survival, in Karonga, Malawi. Open AIDS J.

[10] Ferro-Luzzi A, James WP (1996). Adult malnutrition: simple assessment techniques for use in emergencies. Br J Nutr.

[11] Soeters PB, Schols AM (2009). Advances in understanding and assessing malnutrition. Curr Opin Clin Nutr Metab Care.

[12] Johannessen A, Naman E, Ngowi BJ, Sandvik L, Matee MI, Aglen HE (2008). Predictors of mortality in HIV-infected patients starting antiretroviral therapy in a rural hospital in Tanzania. BMC Infect Dis.

[13] Madec Y, Szumilin E, Genevier C, Ferradini L, Balkan S, Pujades M (2009). Weight gain at three months of antiretroviral therapy is strongly associated with survival: evidence from two developing countries. Aids.

[14] Argemi X, Dara S, You S, Mattei JF, Courpotin C, Simon B (2012). Impact of malnutrition and social determinants on survival of HIV-infected adults starting antiretroviral therapy in resource-limited settings. AIDS.

[15] Sudfeld CR, Isanaka S, Mugusi FM, Aboud S, Wang M, Chalamilla GE (2013). Weight change at 1 mo of antiretroviral therapy and its association with subsequent mortality, morbidity, and CD4 T cell reconstitution in a Tanzanian HIV-infected adult cohort. Am J Clin Nutr.

[16] Sanne I, Mommeja-Marin H, Hinkle J, Bartlett JA, Lederman MM, Maartens G (2005). Severe hepatotoxicity associated with nevirapine use in HIV-infected subjects. J Infect Dis.

[17] Subbaraman R, Chaguturu SK, Mayer KH, Flanigan TP, Kumarasamy N (2007). Adverse effects of highly active antiretroviral therapy in developing countries. Clin Infect Dis.

[18] Munyazesa E, Emile I, Mutimura E, Hoover DR, Shi Q, McGinn AP, et al. Assessment of haematological parameters in HIV-infected and uninfected Rwandan women: A cross-sectional study. *BMJ Open* 2012;2(6):1–8. 10.1136/bmjopen-2012-001600 PMC3533001 23169875

[19] Gibellini D, Clo A, Morini S, Miserocchi A, Ponti C, Re MC (2013). Effects of human immunodeficiency virus on the erythrocyte and megakaryocyte lineages. World J Virol.

[20] Campbell K (2004). Pathophysiology of anaemia. Nurs Times.

[21] May M, Boulle A, Phiri S, Messou E, Myer L, Wood R (2010). Prognosis of patients with HIV-1 infection starting antiretroviral therapy in sub-Saharan Africa: A collaborative analysis of scale-up programmes. The Lancet.

[22] Wester CW, Eden SK, Shepherd BE, Bussmann H, Novitsky V, Samuels DC (2012). Risk factors for symptomatic hyperlactatemia and lactic acidosis among combination antiretroviral therapy-treated adults in Botswana: results from a clinical trial. AIDS Res Hum Retroviruses.

[23] Graham SM, Baeten JM, Richardson BA, Wener MH, Lavreys L, Mandaliya K (2007). A decrease in albumin in early HIV type 1 infection predicts subsequent disease progression. AIDS Res Hum Retroviruses.

[24] Dao CN, Peters PJ, Kiarie JN, Zulu I, Muiruri P, Ong'ech J (2011). Hyponatremia, hypochloremia, and hypoalbuminemia predict an increased risk of mortality during the first year of antiretroviral therapy among HIV-infected Zambian and Kenyan women. AIDS Res Hum Retroviruses.

[25] Sudfeld CR, Isanaka S, Aboud S, Mugusi FM, Wang M, Chalamilla GE (2013). Association of serum albumin concentration with mortality, morbidity, CD4 T-cell reconstitution among tanzanians initiating antiretroviral therapy. J Infect Dis.

[26] Gatta A, Verardo A, Bolognesi M (2012). Hypoalbuminemia. Intern Emerg Med.

[27] Koethe JR, Blevins M, Nyirenda C, Kabagambe EK, Shepherd BE, Wester CW (2011). Nutrition and inflammation serum biomarkers are associated with 12-week mortality among malnourished adults initiating antiretroviral therapy in Zambia. J Int AIDS Soc.

[28] Mehta SH, Astemborski J, Sterling TR, Thomas DL, Vlahov D (2006). Serum albumin as a prognostic indicator for HIV disease progression. AIDS Res Hum Retroviruses.

[29] Kannangai R, Kandathil AJ, Ebenezer DL, Mathai E, Prakash AJ, Abraham OC (2008). Usefulness of alternate prognostic serum and plasma markers for antiretroviral therapy for human immunodeficiency virus type 1 infection. Clin Vaccine Immunol.

[30] Olawumi HO, Olatunji PO (2006). The value of serum albumin in pretreatment assessment and monitoring of therapy in HIV/AIDS patients. HIV Med.

[31] Bocedi A, Notaril S, Narciso P, Bolli A, Fasano M, Ascenzi P (2004). Binding of anti-HIV drugs to human serum albumin. IUBMB Life.

[32] Boffito M, Back DJ, Blaschke TF, Rowland M, Bertz RJ, Gerber JG (2003). Protein binding in antiretroviral therapies. AIDS Res Hum Retroviruses.

[33] Etard JF, Ndiaye I, Thierry-Mieg M, Gueye NF, Gueye PM, Laniece I (2006). Mortality and causes of death in adults receiving highly active antiretroviral therapy in Senegal: a 7-year cohort study. AIDS.

[34] WHO. "Physical status: the use and interpretation of anthropometry. Report of a WHO Expert Committee." World Health Organ Tech Rep Ser. 1995;854:1-452. 8594834

[35] Heimburger D, Shils ME, Shike M, Catharine Ross A, Caballero B, Cousins RJ (2006). Adulthood. Modern Nutrition in Health and Disease.

[36] WHO (2011). Haemoglobin concentrations for the diagnosis of anaemia and assessment of severity.

[37] Johannessen A, Naman E, Gundersen SG, Bruun JN (2011). Antiretroviral treatment reverses HIV-associated anemia in rural Tanzania. BMC Infect Dis.

[38] van Griensven J, Thai S (2011). Predictors of immune recovery and the association with late mortality while on antiretroviral treatment in Cambodia. Trans R Soc Trop Med Hyg.

[39] Li N, Spiegelman D, Drain P, Mwiru RS, Mugusi F, Chalamilla G (2012). Predictors of weight loss after HAART initiation among HIV-infected adults in Tanzania. AIDS.

[40] Ssali F, Stohr W, Munderi P, Reid A, Walker AS, Gibb DM (2006). Prevalence, incidence and predictors of severe anaemia with zidovudine-containing regimens in African adults with HIV infection within the DART trial. Antivir Ther.

[41] Don BR, Kaysen G (2004). Serum albumin: relationship to inflammation and nutrition. Semin Dial.

[42] Dusingize JC, Hoover DR, Shi Q, Mutimura E, Kiefer E, Cohen M (2012). Association of serum albumin with markers of nutritional status among HIV-infected and uninfected Rwandan women. PLoS One.

[43] Seres DS (2005). Surrogate nutrition markers, malnutrition, and adequacy of nutrition support. Nutr Clin Pract.

[44] Mildvan D, Creagh T, Leitz G (2007). Anemia Prevalence Study G. Prevalence of anemia and correlation with biomarkers and specific antiretroviral regimens in 9690 human-immunodeficiency-virus-infected patients: findings of the Anemia Prevalence Study. Curr Med Res Opin.

[45] Anude CJ, Eze E, Onyegbutulem HC, Charurat M, Etiebet MA, Ajayi S, et al. Immuno-virologic outcomes and immuno-virologic discordance among adults alive and on anti-retroviral therapy at 12 months in Nigeria. *BMC Infectious Diseases* 2013;13(1):1–19. 10.1186/1471-2334-13-113 PMC3599241 23452915

[46] Ekouevi DK, Balestre E, Ba-Gomis FO, Eholie SP, Maiga M, Amani-Bosse C (2010). Low retention of HIV-infected patients on antiretroviral therapy in 11 clinical centres in West Africa. Trop Med Int Health.

[47] Assefa Y, Kiflie A, Tesfaye D, Mariam DH, Kloos H, Edwin W (2011). Outcomes of antiretroviral treatment program in Ethiopia: retention of patients in care is a major challenge and varies across health facilities. BMC Health Serv Res.

[48] Ochieng-Ooko V, Ochieng D, Sidle JE, Holdsworth M, Wools-Kaloustian K, Siika AM (2010). Influence of gender on loss to follow-up in a large HIV treatment programme in western Kenya. Bull World Health Organ.

